# DeepGOMeta for functional insights into microbial communities using deep learning-based protein function prediction

**DOI:** 10.1038/s41598-024-82956-w

**Published:** 2024-12-30

**Authors:** Rund Tawfiq, Kexin Niu, Robert Hoehndorf, Maxat Kulmanov

**Affiliations:** 1 KAUST Center of Excellence for Smart Health (KCSH), King Abdullah University of Science and Technology, Thuwal, 23955 Saudi Arabia; 2 Biological and Environmental Sciences & Engineering (BESE) Division, King Abdullah University of Science and Technology, Thuwal, 23955 Saudi Arabia; 3 Computer, Electrical and Mathematical Sciences & Engineering (CEMSE) Division, King Abdullah University of Science and Technology, Thuwal, 23955 Saudi Arabia; 4 SDAIA-KAUST Center of Excellence in Data Science and Artificial Intelligence, King Abdullah University of Science and Technology, Thuwal, 23955 Saudi Arabia; 5 KAUST Center of Excellence for Generative AI, King Abdullah University of Sciene and Technology, 23955 Thuwal, Saudi Arabia

**Keywords:** Protein function, Microbial samples, Metagenomes, Functional clustering, Gene ontology, Machine learning, Protein function predictions, Metagenomics, Microbiome

## Abstract

Analyzing microbial samples remains computationally challenging due to their diversity and complexity. The lack of robust *de novo* protein function prediction methods exacerbates the difficulty in deriving functional insights from these samples. Traditional prediction methods, dependent on homology and sequence similarity, often fail to predict functions for novel proteins and proteins without known homologs. Moreover, most of these methods have been trained on largely eukaryotic data, and have not been evaluated on or applied to microbial datasets. This research introduces DeepGOMeta, a deep learning model designed for protein function prediction as Gene Ontology (GO) terms, trained on a dataset relevant to microbes. The model is applied to diverse microbial datasets to demonstrate its use for gaining biological insights. Data and code are available at https://github.com/bio-ontology-research-group/deepgometa

## Introduction

Protein function prediction has evolved significantly over the past few years, transitioning from reliance on basic sequence alignment to approaches based on machine learning, natural language processing, or analysis of biological networks^1^. Despite these advances, few methods have been developed for and evaluated on metagenome or amplicon sequencing data mainly because there is no “ground truth” unless the methods are applied to “mock communities” which are highly simplified versions of actual microbial communities and not representative of the complexities encountered in real-world cases.

Deep learning methods stand out as promising solutions capable of annotating novel proteins by learning complex patterns within large datasets^2^. Their ability to annotate proteins without prior explicit sequence similarity or homology can potentially overcome the challenges presented by microbial data. However, the applicability of deep learning-based function prediction methods in annotating microbial genomes is hindered by two main limitations: the lack of training on datasets that are representative of microbial communities and the absence of applications on amplicon data and proteins from whole genome sequencing (WGS) reads.

These limitations underscore a significant gap in the field, where even sophisticated approaches like those presented in the Critical Assessment of Function Annotation (CAFA)^3^challenge, and methods such as ProtInfer^4^and SPROF-GO^5^, utilize databases rich in eukaryotic proteins such as SwissProt, overlooking the predominantly prokaryotic nature of metagenomes^6^. While some deep learning methods, such as NetQuilt^7^and DeepSS2GO^8^, have been specifically trained on bacteria and show commendable performance, their training sets focus on well-studied bacterial species and do not incorporate archaea or viruses. Similarly, these deep learning-based methods are not directly applied to datasets that are representative of complex communities and rely on metrics that do not address the biological relevance of the annotations, further increasing the divide between methodological innovation and practical applicability in microbial genomics.

Acknowledging these challenges, we train a deep learning method for the functional annotation of microbial data and explore the biological significance of these annotations. DeepGOMeta incorporates ESM2 (Evolutionary Scale Modeling 2)^9^, a deep learning framework that extracts meaningful features from protein sequences by learning from evolutionary data. We utilize these learned features through ESM2, train on a more representative dataset, and evaluate against state-of-the-art function annotation methods.

## Methods

### Materials and data

#### UniProtKB/Swiss-Prot dataset and gene ontology

We obtained all proteins that were manually curated and reviewed from the UniProtKB/Swiss-Prot Knowledgebase (v2023_03, r28-6–2023)^10^. We further filtered to select for proteins that belong to prokaryotes, archaea and phages, and only kept proteins with experimental functional annotations using evidence codes EXP, IDA, IPI, IMP, IGI, IEP, TAS, IC, HTP, HDA, HMP, HGI, HEP. The dataset contains 10, 107 reviewed and manually annotated proteins.

Metagenomes contain many uncharacterized, novel proteins and in order to evaluate our models on novel proteins, we generated training, validation and testing splits based on sequence similarity. First, we grouped the proteins by their similarity using Diamond (v2.0.9)^11^ (e-value 0.001) and split them into training, validation and testing sets, 81/9/10 %, respectively. This is to ensure that the training and validation set proteins do not have any similar sequences in the training set.

We trained and evaluated a separate model for each of the GO sub-ontologies: Molecular Functions (MFO) of the protein, Biological Processes (BPO) in which the protein participates, and Cellular Components (CCO) in which the protein operates (r2023-01-01)^12^.

To compare our model against other methods, we generated a time-based test set by following the CAFA^3^ challenge time-based approach. We downloaded UniProtKB/Swiss-Prot (v2023_05 r2023-11-08) and extracted newly annotated proteins in this version. Table 1 summarizes the datasets for each sub-ontology.

**Table 1:** Summary of the UniProtKB/Swiss-Prot dataset.

Ontology	GO Terms	Proteins	Train	Valid	Test	Time-based Split
MFO	3,022	6,280	5,297	354	629	30
BPO	4,861	6,620	5,563	453	604	45
CCO	537	4,975	4,174	398	403	26

The table shows for each GO sub-ontology the number of unique GO terms, the total number of proteins, and the number of proteins in training, validation, and testing sets for the UniProtKB/Swiss-Prot dataset. The last column shows the number of proteins for each sub-ontology used in the time-based split.

#### Protein-protein interactions data

For the 10,107 proteins in our dataset, we obtained protein–protein interaction (PPI) data from the STRING (v11.0)^13^ database, which yielded 14,524 interactions. There were 7 different modes of interactions: binding, activation, reaction, catalysis, expression, inhibition, and ptmod.

#### Paired 16S and WGS dataset

We applied our method to generate functional profiles of microbial communities using four publicly available datasets that contain both 16S amplicon data and WGS from the same samples, shown in Table 2. We used two human stool microbiomes: 60 samples from Indian individuals (PRJNA397112) and 60 samples from Cameroonian individuals (WGS: PRJEB27005, 16S: mgp15238)^14,15^, an environmental microbiome: 22 blueberry plant soil samples (WGS: PRJNA484230, 16S: PRJNA389786), and 11 mammalian stool samples (WGS: SRP115632, 16S: SRP115643). The datasets represent a variety of host-associated and environmental microbiomes.

**Table 2:** Descriptions of the paired datasets used for evaluation.

Name	Biome	N	Region	OTUs
India	human stool	60	V3	2077
Cameroon	human stool	60	V5-V6	721
Blueberry	terrestrial	22	V6-V8	1824
Mammalian Stool	mammalian stool	11	V6-V8	420

Biome, number of samples, 16S rRNA gene region and number of identified OTUs for each dataset.

### Baseline and comparison methods

To evaluate our retrained model, we used baseline methods that do not rely on predictions based on sequence similarity, as we aimed to test the predictors on challenging sequences. We used a “naive” classifier leveraging the annotation frequencies within the Gene Ontology (GO) database, a multi-layer perceptron (MLP) model utilizing protein embeddings from the ESM2 model, and several advanced predictive models including DeepGO-PLUS, DeepGOCNN, and DeepGOZero. We also included DiamondScore, which is primarily based on sequence similarity. For the time-based dataset evaluation, we selected several state-of-the-art methods: TALE^16^, SPROF-GO^5^, DeepFRI^17^, DeepGO-SE^18^, NETGO 3.0^19^, and TransFun^20^. We also compared against DiamondScore. We verified that the test protein set in the time-based split was not used for training SPROF-GO, DeepFRI or TALE, but could not verify this for NetGO 3.0 and TransFun due to the unavailability of the training data. Detailed methodologies, including the specific implementations employed in our evaluations, the model versions, and release dates, are documented in the Supplementary Materials section.

### Evaluation

#### Data processing

We analyzed four diverse microbiome datasets, each containing paired 16S rRNA amplicon and WGS data. For the 16S data, we used a Nextflow pipeline employing the RDP classifier (v18) for processing and taxonomic classification available on our GitHub repository (https://github.com/bio-ontology-research-group/16SProcessing). We sourced protein sequences corresponding to the identified bacteria in the RDP database from NCBI and annotated with DeepGOMeta^21,22^. We then used these predicted functions to create two types of functional profiles. We constructed a binary matrix of all the samples and functions in the dataset, where the presence of a function in a sample is represented by 1 and the absence by 0. We also constructed an abundance-weighted matrix for each sample, where we calculated function abundance to provide a quantitative assessment of the functional potential within a microbial sample. The abundance of a function (*A*(*f*) is the sum of the relative abundance of all taxa present in a sample that contain a certain function, given by:

1 $$\begin{aligned} A(f) = \sum _{i=1}^{n} R(t_i) \cdot I(f, t_i) \end{aligned}$$

where *i* is an index representing each taxon, *n* is the total number of taxa in the sample, $R(t_i)$ is the relative abundance of the $i^{th}$ taxon, and $I(f, t_i)$ is an indicator function that equals 1 if the $i^{th}$ taxon contains function *f*, and 0 otherwise.

For WGS data, we used fastp (v0.23.2)^23^for trimming [-q 30]. For host-associated microbiome samples, we used Bowtie2 (v2.5.1)^24^to filter out reads mapping to the host’s reference genome. We then assembled the reads with MEGAHIT (v1.2.9)^25^, predicted protein sequences with prodigal (v2.6.3)^26^, and annotated the predicted proteins using DeepGOMeta. For each sample, we constructed a functional profile by aggregating the functions derived from DeepGOMeta annotations of all proteins present in the sample. We constructed a binary matrix for these results as described above.

**Fig. 1:**
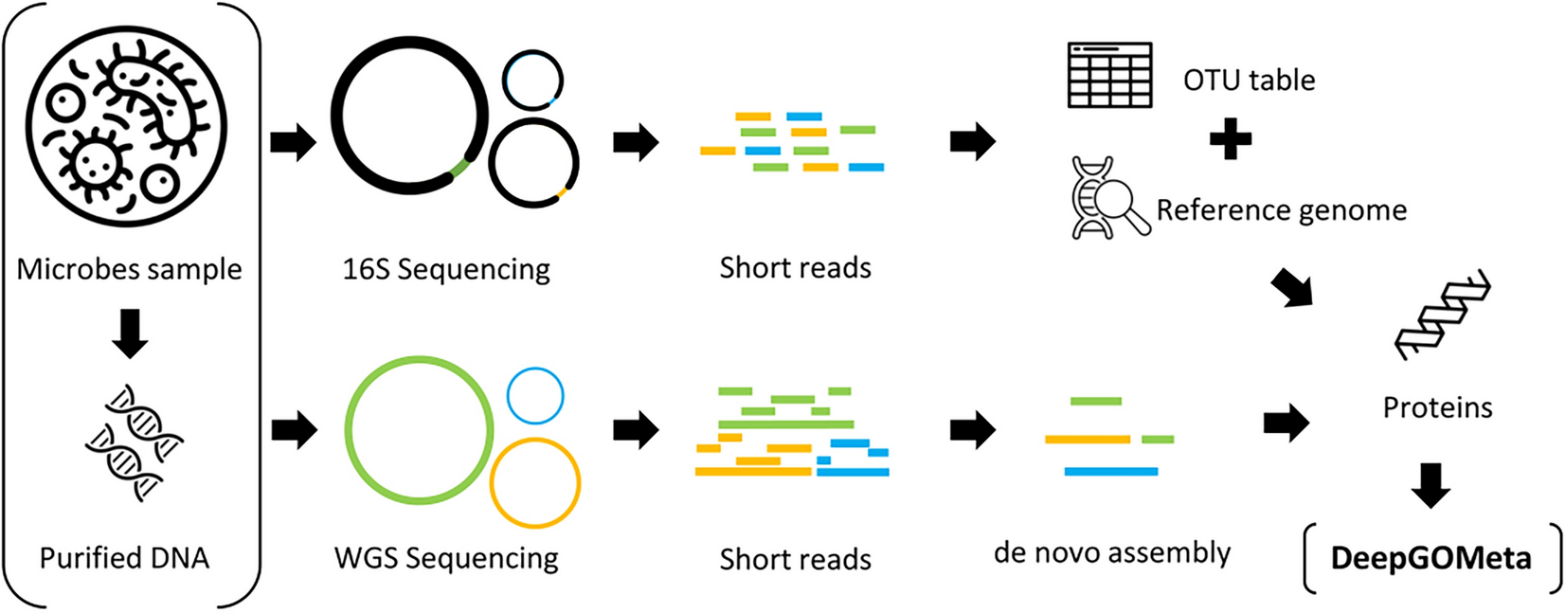
The figure provides an overview of the workflows used to generate functional profiles using DeepGOMeta for amplicon samples and WGS samples.

#### Pathway prediction

PICRUSt2^27^provides the potential functions of microbial communities using 16s rRNA data and a reference genome databases. We used operational taxonomic unit (OTU) tables as the input for PICRUSt2 and focused on MetaCyc^28^ pathways and their abundance scores. We performed Principal Component Analysis (PCA) and k-means clustering to discern patterns within the dataset based on these MetaCyc pathway features. The value of k was determined based on the number of categories within each phenotype. We measured clustering purity based on the true phenotype labels in the datasets (eq. 2).

HUMAnN (The HMP Unified Metabolic Analysis Network) 3.0^29^ efficiently maps metagenomic sequences to a vast database of reference genomes and metabolic pathways. We applied this tool on our benchmarks datasets using WGS data to obtain pathway annotations. We measured clustering purity as described above.

#### Evaluation strategy

For each dataset, we applied PCA and *k*-means clustering to the OTU table containing the relative abundance of bacterial genera, as well as the abundance matrices we constructed using function annotations from each method. The choice of *k* in *k*-means clustering was determined by the number of phenotype categories present for each phenotype under investigation. We calculated clustering purity based on the known phenotype category labels provided in the metadata. Purity assesses the homogeneity of clusters formed by a *k*-means clustering algorithm. We clustered samples based on their bacterial composition and predicted functions, and used purity to evaluate whether samples with same the phenotype are in the same cluster. The Weighted Average Clustering Purity (WACP) formula is given by:

2 $$\begin{aligned} wAvgP = \frac{1}{N} \sum _{i=1}^{N} \left( \frac{\sum _{j=1}^{k} w_j \cdot n_{ij}}{n_i} \right) \end{aligned}$$

where *N* is the total number of data points, *k* is the number of clusters, $n_{ij}$ is the number of data points from cluster *j* that are assigned to cluster *i*, $n_i$ is the total number of data points assigned to cluster *i*, and $w_j$ is the weight associated with cluster *j*.

## Results

### DeepGOMeta

Microbial samples are complex and contain many uncharacterized proteins. Previously, we developed DeepGO-SE^18^to predict protein functions using ESM2^9^ embeddings and approximate semantic entailment. While DeepGO-SE can annotate uncharacterized proteins, it’s trained on all experimentally annotated UniProt-KB/Swissprot proteins, many of the functions it predicts are not relevant to microbiomes and exist only in eukaryotic genomes. Therefore, we trained DeepGOMeta, a specific version of DeepGO-SE, on a dataset of prokaryotic, archaeal and viral proteins with experimental annotations from UniProt-KB/Swissprot. We assessed the model’s performance against other state-of-the-art methods, and applied it to diverse microbial datasets to extract biological insights.

### Incorporating PPIs

Proteins do not function in isolation and PPIs play a significant role in biological processes that take place in the environment. PPI networks also offer a means to reveal functional information for unknown proteins within microbial datasets. In order to test if incorporating PPIs improve protein function prediction, we trained a model which combines PPIs from STRING Database^13^ using Graph Attention Networks. We refer to this model as DeepGOMeta-PPI.

### Evaluation on the similarity-based benchmark

We trained, validated and tested our models for the three sub-ontologies of GO using the UniProtKB/Swiss-Prot dataset split based on sequence similarity (See Methods section). We compared with DiamondScore and four baseline methods that do not rely on sequence similarity: MLP (ESM2), DeepGraphGO, InterPro and Naive.

In the MFO evaluation, DeepGOMeta was outperformed by InterPro in terms of $F_{\max }$ and $S_{\min }$, but performed better than all the other methods in terms of AUPR and term-centric AUC. Combining PPI network features into the model reduced its performance, but was still better than the DeepGraphGO method, which is also based on PPIs (Table 3).

In the BPO evaluation, our model resulted in best $F_{\max }$ of 0.476 which was significantly better (Wilcoxon signed-rank test p-value is $8 \cdot 10^{-37}$) than the second best MLP (ESM2) baseline. Combining PPI networks in DeepGOMeta lead to a slightly lower $F_{\max }$ of 0.469.

In the CCO evaluation, DeepGOMeta achieved the best $F_{\max }$ of 0.739 followed by almost the same performance by MLP(ESM2) baseline. Noticeably, MLP(ESM2) resulted in the best $S_{\min }$. Similarly to MFO and BPO evaluations, combining PPIs did not improve the predictions. DeepGraphGO method resulted in $F_{\max }$ of 0.501, which is slightly better than Naive classifier, and InterPro annotation-based prediction performance was considerably lower than most methods (Table 3).

DiamondScore^30^ was vastly outperformed by all the other methods. Its poor performance may be attributed to its reliance on sequence similarity, which can be limiting for prediction tasks involving proteins with low sequence conservation. This also suggests that methods incorporating sequence or structure embeddings may capture more nuanced relationships beyond sequence similarity.

Using ESM2^9^ embeddings and graph attention mechanisms, we aimed to leverage PPI network contextual information to enrich protein features. However, the sparse interaction data (10,107 proteins, 14,524 interactions, only 1,935 proteins had interactions) introduced noise and did not improve function prediction. Given the sub-optimal performance and sparsity of PPI data, we excluded the DeepGOMeta-PPI model from further evaluation.

**Table 3:** Evaluation results for the sequence-similarity split.

Method	MFO				BPO				CCO			
	$F_{\max }$	$S_{\min }$	AUPR	AUC	$F_{\max }$	$S_{\min }$	AUPR	AUC	$F_{\max }$	$S_{\min }$	AUPR	AUC
Naive	0.291	16.447	0.157	0.500	0.303	25.498	0.199	0.500	0.372	8.032	0.183	0.500
InterPro	**0.548**	**11.309**	0.200	0.663	0.447	20.442	0.248	0.576	0.256	7.257	0.313	0.598
MLP(ESM2)	0.465	12.587	0.432	0.723	0.449	20.095	0.419	0.811	0.738	**3.369**	0.741	0.897
DeepGraphGO	0.315	14.385	0.223	0.456	0.340	22.607	0.283	0.507	0.501	5.218	0.494	0.577
DeepGOMeta	0.468	12.230	**0.449**	**0.874**	**0.476**	**19.394**	**0.462**	**0.847**	**0.739**	3.394	**0.759**	**0.903**
DeepGOMeta-PPI	0.436	12.909	0.392	0.839	0.469	19.715	0.443	0.823	0.706	3.648	0.710	0.869
DiamondScore	0.085	17.202	0.238	0.519	0.091	26.575	0.093	0.507	0.060	8.525	0.114	0.504

This table shows protein-centric$F_{\max }$,$S_{\min }$, and AUPR, and the class-centric average AUC.

### Evaluation and comparison on the time-based split

Microbial data often contains many uncharacterized proteins, so we used a time-based split to ensure that our model is robust and effective in prediction the functions of novel proteins. We compared DeepGOMeta predictions on newly annotated proteins with other state-of-the-art methods that predict functions based on protein language model embeddings and transformer-based deep learning models, including TALE^16^, SPROF-GO^5^, DeepFRI^17^, DeepGO-SE^18^, NetGO 3.0^19^, and TransFun^20^. We also compared these methods with the Naive classifier and InterPro.

We found that DeepGOMeta outperformed all the compared methods in the BPO and CCO evaluations in terms of $S_{\min }$, and CCO in terms of $F_{\max }$. However, it resulted in lower performance than NetGO 3.0 and InterPro in terms of $F_{\max }$ in MFO and lower performance than NetGO 3.0 in terms of AUC in MFO and BPO. DeepFRI outperformed all methods in terms of $S_{\min }$ in the MFO evaluation. DeepGOMeta outperformed DeepGO-SE in BPO and CCO evaluations, but DeepGO-SE had better performance in terms of $S_{\min }$ and AUC in MFO evaluation (Table 4).

Due to the implementation of NetGO 3.0 on the webserver, and the unavailability of the training dataset of TransFun, it is important to note that we cannot exclude the possibility that these methods were trained on the proteins used for testing in the time-based split.

**Table 4:** Evaluation results for the time-based split.

Method	MFO			BPO			CCO		
	$F_{\max }$	$S_{\min }$	AUC	$F_{\max }$	$S_{\min }$	AUC	$F_{\max }$	$S_{\min }$	AUC
Naive	0.158	15.152	0.500	0.143	28.159	0.500	0.451	9.392	0.500
InterPro	**0.447**	11.571	0.603	0.114	27.570	0.511	0.335	7.142	0.602
DeepGO-SE	0.369	11.569	0.613	0.371	19.704	0.592	0.696	5.252	0.713
SPROF-GO	0.383	11.299	0.740	0.422	19.035	0.718	0.661	5.427	0.750
TALE	0.229	13.332	0.662	0.283	22.687	0.630	0.653	6.579	0.620
DeepFRI	0.311	**11.295**	0.633	0.332	21.279	0.568	0.510	6.220	0.602
DeepGOMeta	0.382	13.635	0.602	0.431	**18.296**	0.644	**0.749**	**4.497**	0.758
NetGO 3.0	0.423	11.963	**0.806**	**0.436**	23.173	**0.721**	0.720	4.872	**0.790**
TransFun	0.322	13.690	0.712	0.229	26.925	0.628	0.704	6.241	0.670
DiamondScore	0.306	13.842	0.555	0.262	26.722	0.559	0.449	7.937	0.519

### Applications on amplicon and metagenome data

We developed two workflows employing DeepGOMeta for functional characterization of microbial samples from 16S amplicon and WGS reads. Our methodology included generating functional profiles from reference genomes based on OTUs for 16S reads and predicting functions from *de novo* metagenome assemblies for WGS reads, as illustrated in Figure 1. We applied these workflows to paired 16S and WGS datasets from identical samples, and employed our evaluation strategy to assess our method’s efficacy in capturing functionally relevant information. Due to the absence of ground-truth data for microbial functions, we assume that protein functions found in microbial communities are more similar when the microbial communities are from the same environment or share identical phenotypes. This approach allowed us to explore the primary drivers of community composition, focusing on the application of DeepGOMeta for gaining biological insights.

We used DeepGOMeta to construct functional profiles for each sample using reads from both sequencing strategies and compared against taxonomy-based clustering (Table 5). For each dataset, based on DeepGOMeta results, we constructed a binary representation of functions which indicates presence or absence of a function. For 16S data, we also constructed an abundance-weighted matrix, in which each function is assigned a weight (eq. 1).

In certain contexts, DeepGOMeta demonstrated superior performance over OTU-based clustering. Specifically, in 5 out of the 9 phenotypes we analyzed, employing 16S functions (abundance-weighted) proved to be either on par with or more effective than clustering by OTUs. This suggests that DeepGOMeta’s functional profiles can be effective in capturing specific functional attributes that are unique to each phenotype. In some datasets, such as Mammalian Stool and Cameroon (Region, Ethnicity), the functional attributes were more defining than taxonomic composition, suggesting that these community compositions are driven by functions (in contrast to taxa), in line with previous findings of the relationship between host phylogeny and gut microbiome functional differences^31^.

Conversely, in 3 out of 9 phenotypes studies, OTU-based clustering proved more effective. Specifically, in two datasets (Blueberry, India), the location phenotype was better explained by OTU composition than by functions, consistent with previous findings^32^. Interestingly, we found that using 16S functions in a binary format never outperformed the abundance-weighted approach, suggesting its limited efficacy. In the case of WGS functions, this method only took the lead in 1 out of 9 phenotypes, possibly indicating the necessity of weighing functions.

We also compared OTU-based clustering and DeepGOMeta-derived functional profiles with pathway predictions from tools commonly used to annotate microbial data. We generated pathway predictions from WGS data using HUMAnN3^29^and from amplicon data using PICRUSt2^27^. When comparing WGS functions predicted by DeepGOMeta to those of HUMAnN3, we found that the two methods performed equally well in separating the phenotypes in 3 cases, but DeepGOMeta showed superior performance in 4 cases. Notably, HUMAnN3 failed to produce sufficient pathway information for clustering in analyzing the Blueberry dataset.

When comparing 16S functions (abundance-weighted) predicted by DeepGOMeta to those of PICRUSt2, we found that DeepGOMeta better separated the phenotypes in 7 out of 9 cases. While the experiment falls short of comparing the performance of the two function prediction methods, compared to DeepGOMeta, PICRUSt2’s pathway information would be considered limited, as it constitutes only a subset of the predictable functions by DeepGOMeta in the form of BPO predictions. Overall, these results indicated either a lack of strong associations between pathways and phenotypes or limitations of the algorithms/databases used by PICRUSt2 and HUMANn3.

**Table 5:** K-means cluster purity based on genus abundance and function abundance (k = number of true phenotype labels).

Datatype	Mammalian Stool	Blueberry		India		Cameroon			
	Host	Type	Location	Location	Diet	Region	Diet	Sex	Ethnicity
OTUs	0.63	**0.63**	**0.77**	**0.62**	**0.73**	0.41	0.43	**0.61**	0.67
16S Functions (Abundance)	**0.72**	**0.63**	0.68	0.58	**0.73**	**0.44**	0.43	0.56	**0.69**
16S Functions (Binary)	0.64	0.55	0.68	0.58	**0.73**	**0.44**	0.48	0.57	0.67
WGS Functions	0.55	0.55	0.68	0.58	**0.73**	0.41	**0.52**	0.56	**0.69**
Pathways(PICRUSt2)	0.60	0.61	0.67	**0.62**	0.69	0.38	0.48	0.55	0.64
Pathways(Humann3)	0.55	-	-	0.58	**0.73**	0.40	0.48	0.55	0.62

## Discussion

In this study we introduced DeepGOMeta, a retrained version of DeepGO-SE, to overcome the limitations of current methods in their lack of representative training sets and the lack of applications on microbial data. We trained, tested, and evaluated three different models on UniProtKB/Swiss-Prot Knowledgebase proteins that belong to microbes (prokaryotes, archaea, viruses) prevalent in microbial datasets. DeepGOMeta provides function predictions in the form of GO terms, as each of the three models was trained on a distinct GO sub-ontology. DeepGOMeta demonstrates an improvement over similarity-based benchmark methods in most evaluation metrics across the BPO and CCO sub-ontologies, but was outperformed by InterPro in terms of $F_{\max }$ and $S_{\min }$ in MFO. In the comparison using a time-based split, DeepGOMeta outperformed all the compared methods in the BPO and CCO evaluations in terms of $S_{\min }$, and CCO in terms of $F_{\max }$. However, it was outperformed by NetGO 3.0 and InterPro in terms of $F_{\max }$ in MFO, and NetGO 3.0 in terms of AUC in MFO and BPO. We could not verify that the test protein set in the time-based split was not used for training NetGO 3.0 and TransFun.

We demonstrated an application of DeepGOMeta in annotating both amplicon and metagenomic data in diverse datasets using a workflow that we have developed for this purpose. We constructed functional profiles for each sample based on 16S amplicon and WGS data, and compared the clustering of phenotypes against clustering based on taxonomic classification, allowing us to explore the primary drivers of community composition. For some phenotypes, we found that generating functional profiles using 16S amplicon data with DeepGOMeta (abundance-weighted) yields a higher clustering purity than clustering by OTUs, demonstrating cases where phenotypic differences can be attributed to functional variability rather than taxonomic composition. We also observed variability in performance across the different datasets and phenotypes, highlighting that microbial community composition could be driven by functions and/or taxonomy. We also found that DeepGOMeta predictions better separate phenotypes when compared with pathway predictions from PICRUSt2 and HUMANn3.

Looking forward, we propose several avenues for further enhancing the method’s utility. We plan to expand the training data to incorporate eukaryotic microbial genomes for WGS analysis, which will enable a more comprehensive understanding of metagenomic samples in ecosystems where eukaryotes play significant roles. Furthermore, as our observations reveal the sparsity of PPIs in bacteria, we intend to incorporate methods for interaction predictions. Integrating such methods as features within DeepGOMeta could substantially enhance its predictive accuracy and, consequently, our understanding of microbial interactions.

Additionally, we plan to expand our bioinformatics workflow in two ways. First, we aim to explore several ways through which we can assign weights to functions assigned to proteins from WGS data. Second, we would use the predicted functions and interactions to elucidate pathways both within and between organisms. This shift towards unraveling more complex biological processes will facilitate a deeper understanding of the intricate interactions and dependencies within microbial communities.

## Data Availability

Data and code are available at https://github.com/bio-ontology-research-group/deepgometa

## Declarations

### Accession codes

Public data utilized in this study was downloaded from the European Nucleotide Archive with the accession codes PRJNA397112, PRJEB27005, PRJNA484230, PRJNA389786, SRP115632, and SRP115643, and MG-RAST with the accession code mgp15238.
